# Tantalum Particles Induced Cytotoxic and Inflammatory Effects in Human Monocytes

**DOI:** 10.1155/2021/6658498

**Published:** 2021-01-29

**Authors:** Yajie Yang, Yaokun Zhang, Yiyuan Kang, Chen Hu, Yanli Zhang, Huimin Liang, Jie You, Longquan Shao

**Affiliations:** ^1^Department of Stomatology, Nanfang Hospital, Southern Medical University, Guangzhou 510515, China; ^2^Department of Stomatology, The First Affiliated Hospital of Guangzhou University of Chinese Medicine, Guangzhou 510515, China

## Abstract

The aim of this study is to evaluate the biological safety of tantalum (Ta) particles and to further explore the effects of Ta particles on human monocyte toxicity and inflammatory cytokine expression. Human monocyte leukemia (THP-1) cells were cultured with Ta and hydroxyapatite (HA) particles. Cell counting kit-8 method was used to evaluate the cytotoxicity of Ta and HA particles. The apoptosis effects were evaluated by flow cytometry, and the protein expression levels of interleukin-6 (IL-6) and tumor necrosis factor-*α* (TNF-*α*) were evaluated by ELISA. The protein levels of inflammation-related signaling pathways including nuclear factor-kappa B (NF-*κ*B) and extracellular regulated kinase (ERK) were detected by western blotting. The cytotoxicity test showed that the toxicity level of Ta *in vitro* was grade l, which is within the clinically acceptable range. Compared with the HA control, Ta had no significant effect on THP-1 cell apoptosis, IL-6, and TNF-*α* release. The phosphorylated levels of NF-*κ*B and ERK at 3 h in the Ta group were lower than those in the HA and control groups (*P* < 0.001 both). These results reveal Ta particles behave good biosafety properties and provide some new insights for the future clinical use of Ta.

## 1. Introduction

Dental implantation is one of the most effective means of repairing dentition defects and loss [1] and has been widely used in dental clinics. Most dental implants have achieved good function and aesthetic effects [2]. However, previous data have shown that some patients and implants have bone loss with different degrees, which means that there is a risk of implant failure [3, 4]. The most common causes of failure are infection and implant surface preparation [5]. Firstly, peri-implant inflammation is an important cause of implant failure. Many kinds of cells, such as monocytes, lymphocytes, adipocytes, and endothelial cells, secrete inflammatory factors which participate in the progress of inflammation. Among the inflammatory factors, interleukin 6 (IL-6) and tumor necrosis factor-*α* (TNF-*α*) are related to bone resorption at the implant and bone interface. As a classic mediator of inflammation, the nuclear factor-kappa B (NF-*κ*B) pathway also plays an important role in bone resorption [6–9]. Secondly, implant failure could also result from corrosion by fluoride ions in toothpaste, gargling and drinking water, and body fluids. The passivation film titanium dioxide (TiO_2_) on the surface of titanium and titanium alloy implants has insufficient corrosion resistance in fluoride-containing environments. Fluoride ions in the oral environment lead to the destruction of the passivation film, eliminating the connection between the implant and bone [10, 11]. Therefore, it is very important to improve the corrosion resistance and biological properties of dental implants. Research that further elucidates surface modifications of titanium implants to improve the surface and overall properties of these implants could effectively shorten the clinical healing time to achieve early biological stability and maintain long-term stability after functional loading.

Tantalum (Ta) materials have good ductility, high temperature resistance, corrosion resistance, and processing performance [12, 13], and have been widely used in medical, aerospace, electronics, and chemical industries [14–16]. It is reported that Ta materials have stable biological characteristics, and its elastic modulus is similar to that of natural bone [17, 18]. Ta can be used as a coating to modify the surface of titanium implants and can make up for the shortcomings of corrosion resistance and poor wear resistance of titanium alloy and hydroxyapatite (HA) coatings [19–21]. Comparing with conventional titanium surfaces, porous tantalum structures have more porosity and structural irregularities. Thus, greater surface areas in porous tantalum structures are available for cell attachment and proliferation [22]. However, the research on the biological safety of Ta particles is limited and needs further exploration.

The purpose of this study was to evaluate the biological safety of Ta particles and to further explore the effects of Ta particles on human monocyte toxicity, the gene expression levels of inflammatory cytokines, and secretion of NF-*κ*B and extracellular regulated kinase (ERK) pathways to understand the application prospects of Ta materials as implant coatings.

## 2. Materials and Methods

### 2.1. Ta Chemicals

Ta (CAS Number: 7440-25-7) and HA (CAS Number: 12167-74-7) particles were obtained from Sigma-Aldrich (USA): tantalum powder, -325 mesh, 99.9% (metal basis; 99.6% purity includes H, N, C, and O) and HA: calcium phosphate tribasic, 34-40% Ca. These products were bought by Shao Lab (Southern Medical University, China).

### 2.2. Material Acquisition and Characterization

The Ta and HA particles were evenly sprinkled on adhesive paper. Excess powder was blown off of the adhesive paper. Then, the surface was sputtered with gold under vacuum. The images were observed and taken with a scanning electron microscope (SEM, Titan Krios, USA). Particle size of Ta and HA particles in distilled water was measured with a Zetasizer (Nano ZS, Malvern, UK).

### 2.3. Human Monocyte Leukemia (THP-1) Cell Culture and Passage

THP-1 cells were obtained from the Cell Bank of the Shanghai Institutes for Biological Sciences in China (SCSP-567) and cultured in low-sugar DMEM cell culture medium containing 10% FBS and cultured at 37°C and 5% CO_2_ in an incubator. The growth status, density, and color of the culture medium were observed. According to the experimental plan, cells at 1 : 2 or 1 : 3 passages were packed into new culture flasks, and cells in the logarithmic growth phase were used for follow-up experiments.

### 2.4. Surface Area Ratio (SAR)

The surface area ratio (SAR) refers to the ratio of the surface area of the material to the cell surface area. The SAR chosen in our study was 10 : 1. The cell surface area was estimated by the surface of each well in the culture plate. For example, the surface of each well in a 96-well culture plate was 0.32 cm^2^ and that in a 6-well culture plate was 9.6 cm^2^. The specific surface area of Ta particles and HA particles was determined by the nitrogen adsorption method. According to the GB/T13390.2008 standard, the specific surface areas of Ta particles and HA particles were measured to be 1.135 m^2^/g and 10.875 m^2^/g, respectively. The experimental materials were sterilized by cobalt-60 radiation and mixed with cell culture media. 0.00039 Ta particles were added to each well of a 96-well culture plate, and 0.00289 HA particles were added to each well in the HA particle group according to the above SAR. In the 6-well culture plate, 0.00909 Ta particles were added to each well in the particle groups, and 0.08409 HA particles were added to each well in the HA particle group.

### 2.5. Cell Counting Kit-8 (CCK-8)

Three groups were included in our experiment: the blank control group, the Ta particle group, and the HA particle group. THP-1 cells were collected and cultured in 96-well culture plates. After 24 h of cell preculture, the experimental materials, which had been sterilized by cobalt-60 radiation, were added to the culture plate of the experimental group. The SAR of the materials to cells was 10 : 1. Then, the 96-well culture plate was incubated in the incubator. The measurement times were 24, 48, and 72 h. At the measurement times, 100 *μ*l of CCK-8 solution was added to each well and incubated at 37°C for 1 h. The absorbance at 450 nm was measured by a microplate reader (Molecular Devices, USA). Calculation formula is as follows: Relative growth rate (RGR) = [(As − Ab)/(Ac − Ab)] × 100%, where these were the optical density (OD) values of the experimental well, Ac is the OD value of the control well, and Ab is the OD value of the blank control well. Finally, the toxicity level was assessed according to relative growth rate (RGR) (Table 1).

### 2.6. THP-1 Cell Apoptosis

THP-1 cells were cultured with HA or Ta for 24, 48, and 72 h, and Annexin V- and propidium iodide- (PI-) stained single-tube samples and unstained samples were prepared to obtain corrections for the flow cytometry parameters. According to the experimental protocol, HA particles and Ta particles were added to the experimental wells and cultured for 24 h; the morphology of THP-1 cells was observed under a microscope (CKX41, Olympus, Japan) and photographed. For flow cytometry, cells were collected (2000 rpm, 5 min) and suspended with binding buffer. Later, 5 *μ*l of Annexin V-FITC (Annexin V Apoptosis Detection Kit, Sigma, USA) and 5 *μ*l of PI were added and gently mixed. Cells were fluorescently stained for 10 min at room temperature protected from light, and flow cytometry was used to evaluate the fluorescence within 1 h. The excitation wavelength was 488 nm, and the emission wavelength was 530 nm.

### 2.7. Quantitative Reverse Transcription Polymerase Chain Reaction (qRT-PCR)

Total RNA was isolated from cells using TRIzol (Thermo Fisher Scientific, Waltham, MA, USA), and RNA was reverse-transcribed into cDNA by the PrimeScript RT reagent kit (EZBioscience, Roseville, USA). SYBR Green Premix Ex Taq (Takara Bio, Tokyo, Japan) was used for subsequent qRT-PCR amplification on an LC480 system. Glyceraldehyde-3-phosphate dehydrogenase (GAPDH) and U6 were used as internal controls.

### 2.8. Quantification of Inflammatory Cytokines by ELISA

THP-1 cells were cultured with HA or Ta for 12, 24, and 48 h, and the quantifications of inflammatory cytokines including IL-6 and TNF-*α* were achieved with the use of the ELISA kits under the manufacturer's instruction (R&D Systems, Minneapolis, MN, USA). The measurements of concentration are presented in the form of picograms per microliter (pg/ml).

### 2.9. Western Blot Analysis

Each protein sample was successively electrophoresed on a 10% sodium dodecyl sulfate polyacrylamide gel and transferred to polyvinylidene difluoride membranes (Bio-Rad, Hercules, CA, USA), which were blocked with 5% fat-free milk in phosphate-buffered saline. Then, the membranes were incubated with specific diluted primary antibodies (dilution 1 : 1000) at 4°C overnight. The primary antibodies included phosphorylated NF-*κ*B (Cell Signaling Technology, AB_331284, USA), NF-*κ*B (Cell Signaling Technology, AB_10859369, USA), ERK (Cell Signaling Technology, AB_390779, USA), and phosphorylated ERK (Cell Signaling Technology, AB_2315112, USA). Mouse monoclonal anti-GAPDH (1 : 20000, Proteintech, USA) was used as internal control. On the next day, the HRP-conjugated goat anti-rabbit secondary antibody (1 : 3000, Boster, China) was incubated with the films at room temperature for 1 h. The blots were assessed with enhanced chemiluminescence (Tanon 5200, China).

### 2.10. Statistical Analysis

The data were analyzed with SPSS 17.0. The quantitative data were compared with two independent sample *t*-tests between two groups. For comparison of the three groups, a one-way analysis of variance (ANOVA) was used. *P* < 0.05 was considered to indicate a statistically significant difference. All experiments were repeated at least three times, and representative experiments are shown.

## 3. Results

### 3.1. Characteristics of Ta and HA Particles

**Figure 1:**
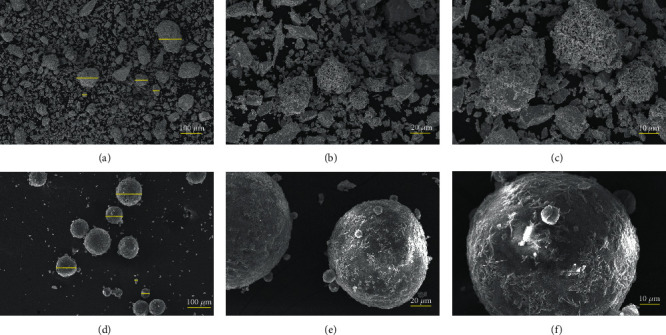
SEM images and particle size distribution of Ta and HA particles. (a–c) Original magnifications ×100, ×500, and ×1000 of Ta particles. (d–f) Original magnifications ×100, ×500, and ×1000 of HA particles.

**Figure 2:**
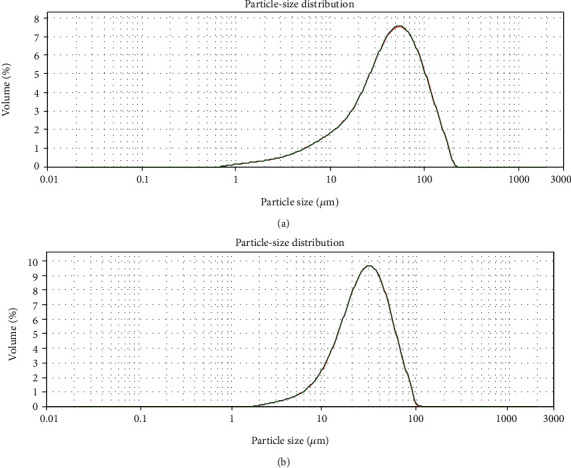
Particle size distribution of (a) Ta and (b) HA particles obtained from DLS measurement.

SEM images showed the sizes and shapes of Ta and HA particles. It can be seen that Ta particles were irregular in shape. The size of HA particles varies. The shape of HA particles was regular and spherical (Figures 1(a)–1(f)). The hydrodynamic size of Ta was mainly between 2 and 200 *μ*m, while the HA particle size is mainly between 1 and 100 *μ*m (Figures 2(a) and 2(b)).

### 3.2. Cytotoxicity Detection of Ta Particles *In Vitro*

**Figure 3:**
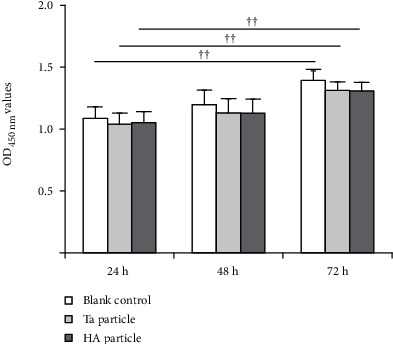
The OD_450 nm_ values of the three groups at different culture times. ^††^*P* < 0.01 comparing with their respective groups at 24 h.

Figure 3 shows the average and standard deviation of OD_450_ in the three groups at different culture times. The OD values of Ta, HA, and blank control groups at 72 h were significantly higher than those at the 24 h (*P* = 0.006, 0.007, and 0.003, respectively). The difference between the HA and Ta groups was not different at each time point.

The relative cell proliferation rates of the HA and Ta groups at the three time points were significantly lower than those of the blank control group (Table 2). According to the criteria listed in Table 1, the toxicity levels of the HA particle and Ta groups at the three time points were grade 1.

### 3.3. Effects of Ta Particle on Apoptosis

**Figure 4:**
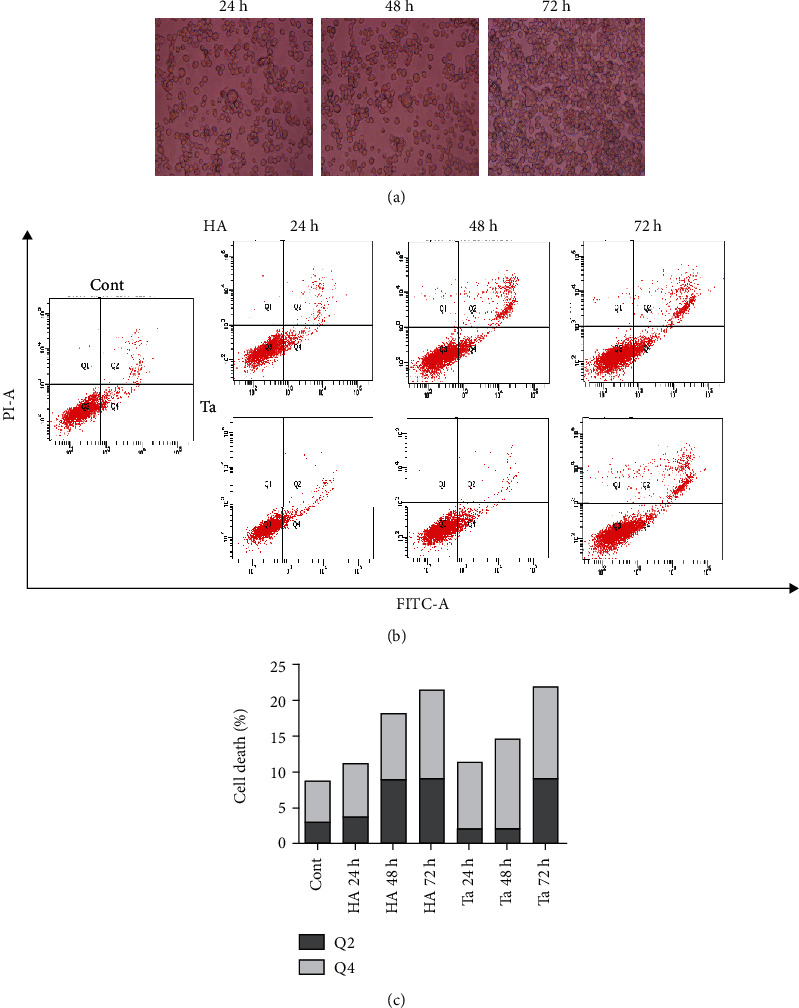
Effects of Ta particle on apoptosis. (a) The number and density of THP-1 cells increased with culture time. (b) Necrotic cells accounted for a small proportion of the total cells. Normal living cells accounted for the main proportion in the three groups, and a small number of apoptotic cells were observed. For apoptotic cells, more cells were in the late stage than those the early stage. (c) The percentage of apoptotic cells increased gradually with different culture times.

THP-1 cells were cultured with Ta particles. With increasing culture time, the number and density of cells increased. The THP-1 cells in the three groups showed little morphological abnormality under the microscope. Few cell fragments and a small number of large, irregularly shaped cells were observed (Figure 4(a)).

According to the results from flow cytometry, the proportion of apoptotic cells in the control group was 8.7%. At 24 h, 48 h, and 72 h in the HA group, the apoptotic cell percentage was 11.1%, 18.2%, and 21.3%, respectively. At 24 h, 48 h, and 72 h in the Ta group, the apoptotic cells accounted for 11.4%, 14.7%, and 22.0%. With longer culture times, the number of apoptotic cells increased gradually (Figures 4(b) and 4(c)).

**Figure 5:**
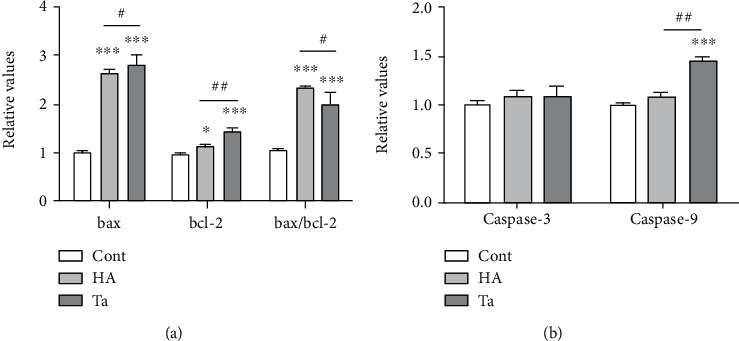
Expression of apoptosis-related genes (bax, bcl-2, bax/bcl-2, caspase-3, and caspase-9) in the control, HA, and Ta groups. (a) The expression of the apoptosis-related genes bax and bcl-2 was significantly upregulated in the HA and Ta groups compared with the control group, and the ratio of bax to bcl-2 was more than twice as high as that in the control group. Both the bax and bcl-2 gene expression levels in the Ta group were significantly higher than those in the HA group, but the bax/bcl-2 ratio in the Ta group was significantly lower than that in the HA group. (b) No significant change was found in the gene expression of caspase-3, but the increase in the gene expression level of caspase-9 suggested that Ta could induce cells to enter the mitochondrion-mediated apoptotic process by activating the caspase-9 pathway on the mitochondrial membrane at this concentration. ^∗^*P* < 0.05 comparing with the blank control group; ^∗∗∗^*P* < 0.001 comparing with the blank control group. ^#^*P* < 0.05 significant difference between the Ta and HA groups; ^##^*P* < 0.01 significant difference between the Ta and HA groups; ^###^*P* < 0.001 significant difference between the Ta and HA groups.

Both the bax and bcl-2 gene expression levels in the Ta groups were significantly higher than those in the HA group (*P* = 0.034 and 0.006, respectively), but the bax/bcl-2 ratio in the Ta group was significantly lower than that in the HA group (*P* = 0.025) (Figure 5(a)). At the same time, no significant change (*P* = 0.456) was found in the expression of the apoptosis-related gene caspase-3, but the increase (*P* = 0.008) in the gene expression level of caspase-9 suggested that Ta could induce cells to enter the mitochondrion-mediated apoptotic process by activating the caspase-9 pathway on the mitochondrial membrane at this concentration (Figure 5(b)).

### 3.4. Effects of Ta Particles on the Protein Level of IL-6 and TNF-*α* in Human Monocytes

**Figure 6:**
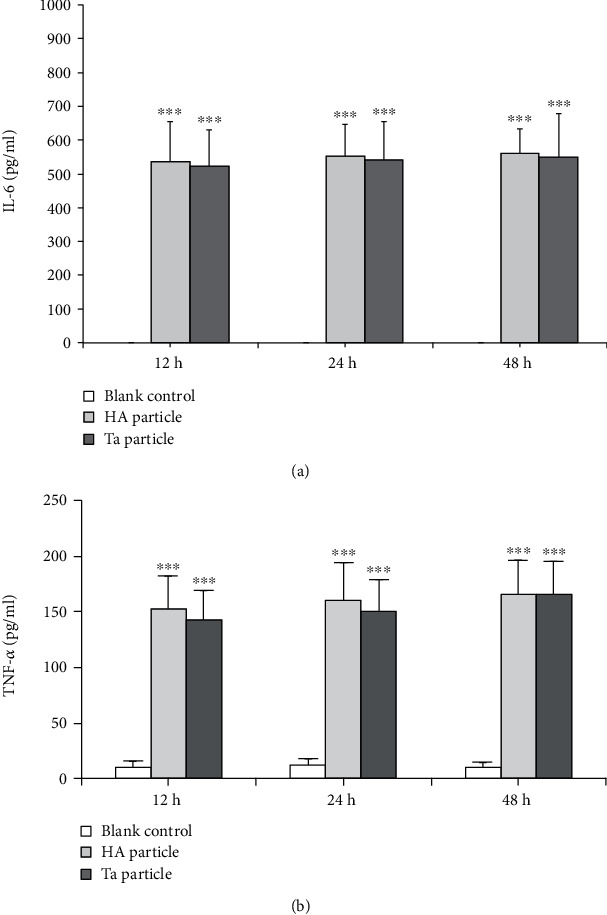
Effects of Ta particles on the secretion of IL-6 and TNF-*α* by human monocytes at different culture times. The expression levels of (a) IL-6 and (b) TNF-*α* in THP-1 cells of the Ta particle group and the HA particle group were significantly higher than those of the blank control group, and there were significant differences. With increased time, the expression levels of IL-6 and TNF-*α* in the Ta particle group and HA particle group showed an increasing trend, but there was no significant difference. ^∗∗∗^*P* < 0.001 comparing with the blank group.

The protein expression levels of IL-6 (Figure 6(a)) and TNF-*α* (Figure 6(b)) in THP-1 cells of the Ta group and the HA group were significantly higher than those of the control group (*P* < 0.001 all), and there were no significant differences between the Ta and HA groups (*P* = 0.740, 0.784, and 0.785 for IL-6, and *P* = 0.349, 0.377, and 0.487 for TNF-*α*).

### 3.5. Effects of Ta Particles on the Expression of NF-*κ*B and ERK in Human Monocytes

**Figure 7:**
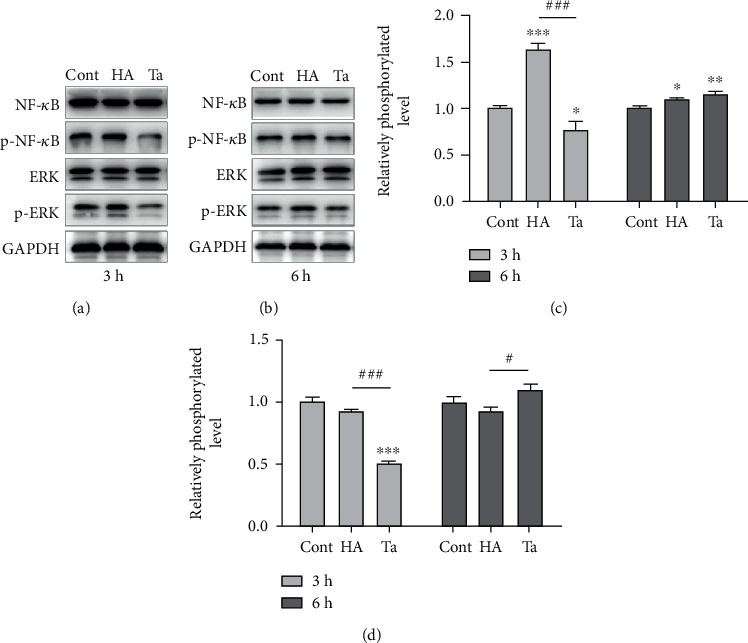
Effects of Ta and HA particles on the expression of NF-*κ*B and ERK in human monocytes after 3 h (a) and 6 h (b) treatment. (c, d) The phosphorylation levels of NF-*κ*B and ERK protein are calculated with ImageJ. ^∗^*P* < 0.05 comparing with the blank control group; ^∗∗^*P* < 0.01 comparing with the blank control group; ^∗∗∗^*P* < 0.001 comparing with the blank control group. ^#^*P* < 0.05 significant difference between the Ta and HA groups; ^###^*P* < 0.001 significant difference between the Ta and HA groups.

The expression levels of NF-*κ*B and ERK in THP-1 cells in the Ta and HA particle groups were also evaluated. After 3 h treatment, the phosphorylation levels of NF-*κ*B and ERK in the Ta particle group were significantly lower than those in the blank (*P* = 0.033 and <0.001, respectively) and HA particle groups (*P* < 0.001 both) (Figure 6). After 6 h treatment, there was no significant difference in the expression of NF-*κ*B between the HA and Ta groups (*P* = 0.280). The ERK protein level in the Ta group is significantly higher than in the HA group (*P* = 0.040) (Figure 7).

## 4. Discussion

HA coating is widely used on dental implant surfaces to form an osteoinductive biological coating [23, 24]. However, HA has two major drawbacks. Firstly, because of the low crystallinity, the coating degrades and dissolves easily in body fluid, decreasing the implant life. Secondly, the thermal expansion coefficients of the HA coating and the implant matrix are not matched, and the weak bonding strength with titanium alloy may result in early exfoliation [25, 26]. Therefore, searching for a better biocoating material has become a hot topic in the research of implant coating materials. Ta is dark gray with a melting point of 2996°C and a relative density of 17.10. The good corrosion resistance and wear resistance of Ta provide broad prospects for the development of Ta biomaterials [27]. Ta can be used as a coating to modify the surface of titanium implants and can overcome the shortcomings of corrosion resistance and poor wear resistance of a titanium alloy with an HA coating [19]. With the progress in implant surface research and considering the limitations of porous tantalum in the application of orthopedic prosthesis surfaces, researchers have reconsidered whether Ta can be used as an implant coating material. More evidence has shown porous tantalum has potential advantages as dental implant coating material comparing with conventional Ti [16, 22, 28]. However, little research has been conducted on the biological safety of Ta as a dental implant coating. Therefore, our study is aimed at investigating the biological safety of Ta material compared with HA material.

SEM showed that the size of Ta particles and HA particles varied. Therefore, the surface area of the experimental material may be different even when the experimental materials have the same quality. The contact area of the experimental materials with the cells and the culture medium largely affects the degree to which experimental materials affect cells biologically. In this study, the experimental materials and cells were cultured at a SAR of 10 : 1 to avoid the effect of surface contact area on experimental reliability. For the toxicity test of Ta particles, the method of culturing cells directly with sterilized particles was used [29, 30]. Compared with traditional MTT assays, the CCK-8 test has the advantages of simpler operation, higher accuracy, better repeatability, and higher sensitivity [31]. The cytotoxicity test *in vitro* showed that the toxicity of Ta particles was grade 1, which is acceptable in the clinic. However, the *in vivo* experiments about the long-term biological safety are still needed.

In this study, we found that the apoptosis rate was not significantly higher at 48 h than at 24 h in each group. However, the apoptosis rate increased significantly as the culture time with the particles increased to 72 h. However, there was no significant difference in the apoptosis rate between the Ta particle group and the HA particle group. Considering the results of the cytotoxicity test *in vitro* and the actual state of cells observed under the microscope, the apoptosis rate of cells cultured for 72 h increased significantly. This increase may be due to the high cell density, the relative shrinkage of the living environment, the limited nutrient content of the culture medium, and the stimulation of materials to a certain extent, resulting in selective apoptosis of cells. This result was consistent with Lemaire et al.'s research [32]. As a traditional coating material for dental implants, HA is widely recognized as a type of bioactive material with good biocompatibility and bone conductivity. A large number of studies have reported that HA has good biocompatibility and can meet the requirements of implant materials, which is consistent with the results of our study [33, 34]. Plasma sprayed HA-coated implants are commonly used. Producing an HA coating with this method results in an uneven density, different crystallinity, and poor long-term stability. The less crystalline HA is, the lower the solubility of the coating is. This conclusion has been verified by other studies [35–37]. Dissolution of HA coatings is the focus of controversy over the long-term effect of HA coating implants because the dissolution is related to peri-implant inflammation, which can lead to the loss of osseointegration and ultimately the loss of implants. Therefore, finding a more stable coating material with good biocompatibility is urgently needed. As a new type of coating material, Ta metal, including the biocompatibility of Ta metal, has emerged as a research focus. The apoptosis rate of cells cultured with Ta particles for 24 and 48 h was not significantly different from that of the blank control group and the HA particle group. The apoptosis rate was only 22.1 ± 1.2% after 72 h of culture with the particles, which is acceptable in the clinic. The *in vitro* results showed that the expression of genes related to cytotoxicity and apoptosis in the Ta particle group was similar to that in the HA particle group. It is preliminarily believed that Ta particles have good biocompatibility and could meet the requirements for a dental implant coating.

The results of this study showed that the expression levels of IL-6 and TNF-*α* increased with incubation time, but the trend was not obvious, and there was no significant difference between the different times. This study also showed that the presence of Ta particles with THP-1 cells during the incubation period can basically maintain a stable stimulating effect on THP-1 cells to secrete inflammatory factors including IL-6 and TNF-*α*. No significant difference was observed between the HA and Ta groups at different times. The inflammation-stimulating effect in both groups is relatively mild. The longer-term inflammation-stimulating effects of Ta particles need further study. The results of this study are consistent with those of some histological studies showing that there are no signs of inflammation in soft tissues around Ta [18].

We further examined the levels of NF-*κ*B and ERK protein expressed in the three groups of cells at 3 and 6 h, as well as their phosphorylation levels. The results showed that the levels of phosphorylated NF-*κ*B at 3 h in the Ta group were lower than those in the HA and control groups, and the levels of phosphorylated NF-*κ*B at 6 h in the Ta and HA groups were significantly higher than those in the control group. Similarly, the level of phosphorylated ERK in the Ta group is significantly lower than in the HA group at 3 h, but higher at 6 h. The results confirmed the inhibitory effect of Ta on the phosphorylation of NF-kappa B and ERK in the early 3 hours, but increased after 6 hours. Ta material could stimulate human monocytes at early stage to become less inflammatory, which might be beneficial for remodeling the bone tissue around implants.

## 5. Conclusions

Compared with HA particles, Ta particles exert a similar effect and even exhibit certain advantages in terms of apoptosis and secretion of inflammatory factors. Overall, Ta showed less cytotoxicity, apoptosis induction, and secretion of inflammatory factors than HA did. This study preliminarily revealed that Ta material has a good biosafety profile, which can provide a basis for the clinical use of Ta.

## Figures and Tables

**Table 1:** Standard of the cytotoxicity grade.

Relative growth rate (%)	Cytotoxicity grade	Assessment
≥100	0	No
75~99	1	No
50759	2	Mild
25~49	3	Moderate
1~24	4	Moderate
0	5	Severe

**Table 2:** The relative growth rate (RGR) and cytotoxicity level in the three groups.

Groups	24 h		48 h		72 h	
	Growth rate (%)	Grade	Growth rate (%)	Grade	Growth rate (%)	Grade
Blank control	100.0	0	100.0	0	100.0	0
Ta particle	95.8^∗^	1	94.5^∗^	1	94.1^∗^	1
HA particle	96.9^∗^	1	94.2^∗^	1	93.9^∗^	1

^∗^Significant difference comparing with the blank control group (*P* < 0.05).

## Data Availability

The data used to support the findings of this study are available from the corresponding author upon request.

## Abbreviations

IL-6: Interleukin 6
TNF-*α*: Tumor necrosis factor-*α*
Ta: Tantalum
HA: Hydroxyapatite
NF-*κ*B: Nuclear factor-kappa B
ERK: Extracellular regulated kinase
TiO_2_: Titanium dioxide
SEM: Scanning electron microscope
SAR: Surface area ratio
CCK-8: Cell counting kit-8
OD: Optical density
RGR: Relative growth rate
PI: Propidium iodide
qRT-PCR: Quantitative reverse transcription polymerase chain reaction
GAPDH: Glyceraldehyde-3-phosphate dehydrogenase
pg/ml: Picograms per microliter
ANOVA: Analysis of variance.
